# Lymph Nodes Involvement and Lymphadenectomy in Thymic Tumors: Tentative Answers for Unsolved Questions

**DOI:** 10.3390/cancers13205085

**Published:** 2021-10-11

**Authors:** Debora Brascia, Angela De Palma, Marcella Schiavone, Giulia De Iaco, Francesca Signore, Teodora Panza, Doroty Sampietro, Gianluca Di Milo, Mariangela Valentini, Salvatore Pisconti, Giuseppe Marulli

**Affiliations:** 1 Thoracic Surgery Unit, Department of Emergency and Organ Transplantation, University Hospital of Bari, 70121 Bari, Italy; deborabrascia@gmail.com (D.B.); angela.depalma@uniba.it (A.D.P.); marcella.schiavone@gmail.com (M.S.); g.deiaco24@gmail.com (G.D.I.); f.signore1@studenti.uniba.it (F.S.); dorapanza91@gmail.com (T.P.); dorotysampietro@gmail.com (D.S.); gianluca_dimilo.546365@unifg.it (G.D.M.); mariangelavalentini88@gmail.com (M.V.); 2 Medical Oncology Department, San Giuseppe Moscati Hospital, 74121 Taranto, Italy; salvatorepisconti@hotmail.it

**Keywords:** lymph node dissection, thymoma, thymic carcinoma, NETTs

## Abstract

**Simple Summary:**

The standard treatment for thymic tumors is radical thymectomy with *en bloc* resection of the surrounding tissue for early stages, while multimodality therapy has to be considered in the advanced stages. Due to the rarity of nodal metastases in thymic tumors, little attention has been paid to their incidence, pattern and prognostic significance and, consequently, no standard nodal mapping or consensus on lymph node dissection has currently been established. Moreover, no data indicate which subgroup of patients would be appropriate candidates for lymph node dissection D or the extent to which lymph nodes should be harvested. The aim of this review is to collect evidence from the useful literature to help physicians in designing the best surgical procedure when dealing with thymic malignancies and to plan the best multidisciplinary strategy in case of advanced stage thymic tumors.

**Abstract:**

Thymic tumors are the most common primary neoplasms of the anterior mediastinum, although, when compared with the entire thoracic malignancies, they are still rare. Few studies addressed the questions about lymph node involvement pattern in thymic neoplasms, about which subgroup of patients would be appropriate candidates for lymph node dissection or about the extent of lymphadenectomy or which lymph nodes should be harvested. The aim of this review is to collect evidence from the literature to help physicians in designing the best surgical procedure when dealing with thymic malignancies. A literature review was performed through PubMed and Scopus in May 2021 to identify any study published in the last 20 years evaluating the frequency and the extent of lymph node dissection for thymic tumors, its impact on prognosis and on postoperative management. Fifteen studies met the inclusion criteria and were included in this review, with a total of 9452 patients with thymic cancers; lymph node metastases were found in 976 (10.3%) patients in total. The current literature is heterogeneous in the classification and reporting of lymph node metastases in thymic carcinoma, and data are hardly comparable. Surgical treatment should be guided by the few literature-based pieces of evidence and by the experience of the physicians.

## 1. Introduction

Thymic tumors are the most common primary neoplasms of the anterior mediastinum, although, when compared with the entire thoracic malignancies, they are still rare. Thymomas represent 90% of thymic neoplasms, accounting for about 0.2 to 1.5% of all cancers; the remaining 10% includes thymic carcinoma (TC), thymic neuroendocrine tumors (NETTs) and lymphomas [1]. 

Standard treatment for early stage thymic malignancies is radical thymectomy with *en bloc* resection of the surrounding thymic tissue, while multimodality therapy should be used for the advanced stages. It is notable that while nodal metastases are rarely seen in thymomas, they are not so uncommon in thymic carcinomas and NETTs, although their incidence differs widely in the literature [2,3,4,5,6,7,8]. Historically, the prevalence of lymph nodes involvement has been described as ranging from 1.8 to 5.1% in thymomas and from 20 to 33.5% in thymic carcinomas and NETTs, but these rates could be underestimated because lymphadenectomy is rarely performed by most institutions [3,6,7,8]. Due to their rarity, little attention has been paid to the incidence, the pattern and prognostic significance of lymph node (LN) metastasis in thymic malignancies and, consequently, no standard nodal mapping or consensus on lymph node dissection (LND) has been established yet. Here comes the question whether to perform lymphadenectomy and how it could impact on prognosis and improve disease control. 

Clinical staging of thymic tumors has been proposed and revised since the 1970s [9,10,11], and Masaoka-Koga staging [12] is still the most popular classification that emphasizes the local invasion of thymoma, while grouping all hematogenous and lymphogenous metastasis as stage IVB. It appears clear that this classification precludes an accurate stratification of patients, especially in carcinomas and NETTs that are affected by high rates of local and distant metastasis. For this reason, since the 1990s, lymph node staging for thymic malignancies has been proposed and updated many times, starting from the first suggested by Yamakawa et al. [13] in 1991, who defined the following three lymph node groups: perithymic as N1, intrathoracic as N2, and extrathoracic as N3. As the role of TNM classification was established for various tumors, a TNM-based stage classification was proposed for thymic tumors on the basis of the Masaoka classification, while including a nodal classification according to the International Thymic Malignancies Interest Group (ITMIG)/IASLC thymic nodal map [14,15,16,17,18]. This nodal map involves the following two nodal compartments: the anterior (N1), including the low anterior cervical, perithymic, prevascular, para-aortic and supradiaphragmatic nodes, and the deep intrathoracic one (N2), including the lower jugular, supraclavicular, internal mammary, paratracheal, subaortic, subcarinal and hilar nodes. 

Although lymph node dissection is now a pivotal part of oncologic surgery for most thoracic malignancies, it is not considered as a standard procedure in thymic cancer surgery yet and the role of LND remains controversial because the actual incidence of lymph node metastasis is well known and the therapeutic role of LND in thymic malignancies has not been established. Due to the lack of knowledge on lymph node pattern in thymic neoplasms, there are no data indicating which subgroup of patients would be appropriate candidates for LND or the extent to which lymph nodes should be harvested, yet. The aim of this review is to collect evidence from the useful literature to help physicians in designing the best surgical procedure when dealing with thymic malignancies and to plan the best multidisciplinary strategy in case of advanced stage thymic tumors.

## 2. Methods

The present systematic review is registered in the International prospective register of systematic reviews PROSPERO with the reference code CRD42021272498. 

### 2.1. Search Strategy

The guidelines for Preferred Reporting Items for Systematic reviews and Meta-Analyses (PRISMA) were applied [19]. A literature review was performed through PubMed and Scopus in May 2021 to identify any study published in the last 20 years evaluating the frequency and the extent of lymph node dissection for thymic tumors, its impact on prognosis and on the postoperative management. The retrieval terms used were “thymic tumors” combined with “lymph node” and “dissection” OR “surgery” OR “lymphadenectomy”. Once the abstracts of potentially relevant studies were scrutinized, each study was independently evaluated by two coauthors (D.B. and A.D.P) for inclusion or exclusion from this analysis. 

Inclusion criteria were as follows: studies describing a lymphadenectomy (of at least one lymph node) during surgery for thymic tumors, data separately reported for histological type of thymic neoplasms and prevalence of patients with lymph node metastases reported or easily evaluable. Articles were ineligible for study if they (1) reported ambiguous or inaccurate data, (2) included pediatric patients, (3) were only imaging studies, case reports, conference abstracts and reviews and (4) were article published in a non-English language.

### 2.2. Data Extraction

Data were independently collected from the retrieved articles by two investigators (D.B. and A.D.P) and checked by two investigators (M.S. and G.M.). No attempt was made to obtain specific or missing data from the authors. When available, the following data were extracted: first author, year of publication, country, type of study, number of patients, type of tumor, overall percentage of positive lymph nodes, incidence of lymph node metastases per histological type, prevalence of lymph node metastases per lymph node station, number of dissected lymph nodes and survival rates. The primary endpoint was the rate of lymph node metastases in thymic tumors. The quality of the included studies was assessed by two investigators (M.S and G.D.I) using the National Heart, Blood and Lung Institute (NHBLI) criteria for study quality assessment of case–control series (https://www.nhlbi.nih.gov/health-pro/guidelines/in-develop/cardiovascular-risk-reduction/tools/case_series; accessed on 10 January 2021) (Table S1). 

### 2.3. Statistical Analysis

Descriptive statistics summarized the characteristics of included studies, patient characteristics and the outcomes of each included study. Not all studies used the same classification or definition for lymph node stations, and not all of them reported data on survival. Moreover, data on survival were not homogeneous since the population from each study differed widely, mainly because of the different histological types taken into consideration. Some of the studies also included patients in which an intentional lymphadenectomy was performed; only these data were collected for the purpose of this analysis. Finally, studies describing the lymphatic distribution pattern according to the pT-stage were pooled and described separately.

## 3. Results

**Figure 1:**
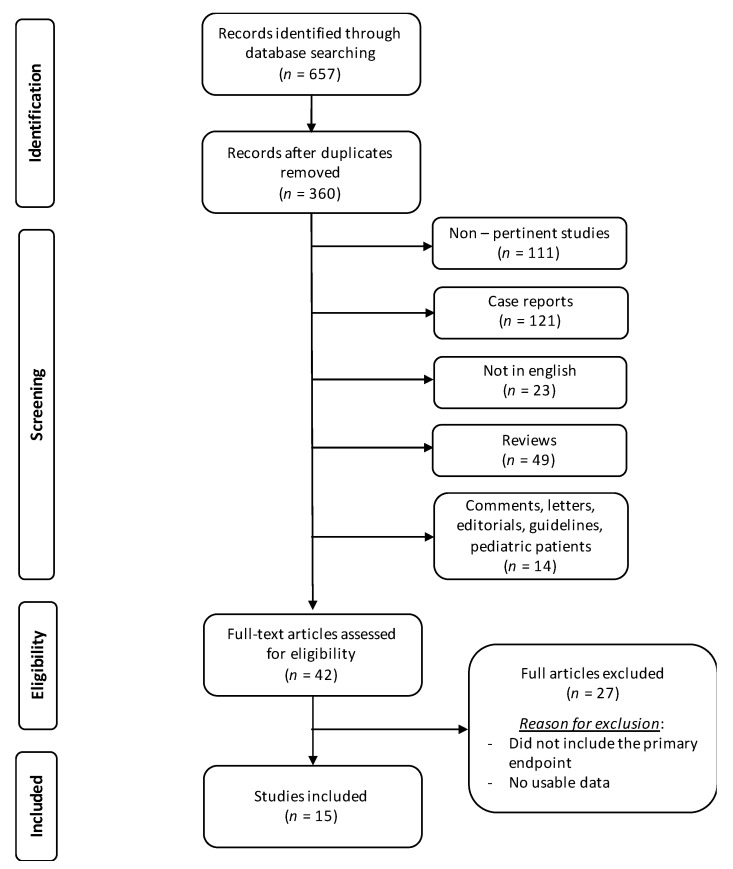
Literature search flowchart.

Literature search and study selection are shown in Figure 1.

Fifteen studies met the inclusion criteria and were included in this review [2,3,4,6,7,8,20,21,22,23,24,25,26,27,28]. Characteristics of the included studies are presented in Table 1.

A total of 9452 patients were evaluated, including 6327 (66.9%) with thymoma, 2450 (25.9%) with thymic carcinoma and 675 (7.1%) with thymic neuroendocrine tumor. Lymph node metastasis was found in 976 (10.3%) patients in total. Lymph node metastases were found in 206 (3.3%) patients with thymoma, 457 (18.6%) patients with thymic carcinoma and 189 (28%) patients with NETTs.

### 3.1. Survival Rates According to the LN Involvement

Five studies [2,6,7,26,27] reported data on survival for both N0 and N+ patients with thymic cancers (Table 2).

All these studies proved a significant difference when comparing the two groups of patients. Almost no study reported data on survival stratified by histological type.

### 3.2. Distribution of LN Metastases in Relation to pT-Stage

Five studies [2,4,21,22,25] stratified the prevalence of nodal metastases per pT-stage.

Table 3 shows the rate of patients with lymph node metastases per histologic type, divided into four groups according to the following pT stages: pT1, T2, T3 and T4. As the T-stage increases, the prevalence of lymph node metastases becomes higher.

## 4. Discussion

Lymph node dissection is now a pivotal part of oncologic surgery; when dealing with thymic malignancies, it is not considered as a standard procedure yet and the role of LND remains controversial. The purpose of this review is to collect the few data published in the last 20 years with the aim of guiding clinicians to design the best surgical procedure and to plan the best multidisciplinary strategy in case of advanced stage thymic tumors by following the existing evidence.

### 4.1. Lymph Node Metastasis in Thymic Tumors: Incidence and Prognosis

Most of the studies in this field do not contain data on lymph node metastasis, since lymph node sampling is not routinely performed during thymoma resection; thus, data on nodal metastases in patients with thymic malignancies are sparse (Table 1). A leading role on this topic has been a study by Kondo et al. [3], who, for the first time in 2003, emphasized the necessity to analyze the pattern of lymphogenous metastasis collecting data from 1320 patients with thymic tumors. The incidence of nodal metastases was 1.8, 26.8 and 27.5%, respectively, in thymomas, thymic carcinomas and NETTs. Five-years survival was strictly dependent on the progression of N factor both in thymomas (95.6% in N0, 61.5% in N1, 20% in N2) and thymic carcinoma (56.0% in N0, 42.1% in N1, 29.3% in N2 and 18.8% in N3) and multivariate analysis showed that lymph node metastases were independent predictors of survival in both groups (*p* = 0.053 and *p* = 0.001). Table 1 resumes data from all the available studies over the last twenty years providing information about LND in thymic cancers; the incidence of LN metastasis in thymoma varies from 0.5 to 13.3%, and in carcinoma varies from 7.9 to 33.5%, while in NETTs it varies from 5.6 to 62.3%. One of the reasons explaining the wide variety of incidence rate across studies, could be the large heterogeneity of the stage, histology and size of tumors among the patients enrolled; additionally, the different policy regarding LND (i.e., intentional standard systematic, locoregional, oriented or “on demand” on the basis of preoperative imaging or intraoperative findings) has increased the bias due to the selection of patients, thus not allowing a comparison between studies. In a study by Weksler et al. [7], in fact, the high rate of LN metastasis in thymomas could be explained by the fact that more than one-half of the tumors in their cohort were Masaoka stage > III. In all the studies, data about survival and freedom from recurrence (FFR) between N0 and N+ patients in all histologic groups significantly differed. In particular, studies by Weksler et al. [6,7] showed the presence of nodal metastases to be an important adverse prognostic factor in patients with thymoma, doubling the risk of death compared with patients who were node negative and in carcinomas and NETTs, increasing the risk of death by approximately threefold. A more recent study by Hwang et al. [8], collecting data from 131 thymic malignancies, confirmed this trend and they proved a five-years FFR rate to be significantly worse in N1/N2 than in N0 (38.5% vs. 87.9%; *p* < 0.001).

**Central message:** lymph node metastases seem to have higher incidence rates in TC and NETTs rather than in thymoma; moreover, these rates differ a lot in the available studies because of the large heterogeneity of the population analyzed. In all cases, the presence of N1/2 involvement has proven to impact negatively on patients in terms of survival and FFR.

### 4.2. Lymph Node Dissection in Thymic Tumors: Is There a Role?

Lymph node dissection is an important prognostic factor in oncological surgery, allowing an accurate tumor staging to plan the postoperative management of patients. Due to insufficient evidence on the role of LND in thymic cancers, whether routine LND is necessary or not remains unclear and whether it could provide a benefit in survival or recurrence has not been documented yet [23]. If the impact of LN metastasis on survival has been previously discussed, nothing has been said about the impact of intentional LND on prognosis. Interestingly, a unique prospective study by Fang et al. [22] involving patients from 15 centers in the ChART group proved rates of LN metastasis to be much higher when compared with a previously reported ChART retrospective study (overall incidence rates 5.5% vs. 2.2%) [2]. This is of paramount importance, since it has suggested for the first time that intentional lymph node retrieval would increase the yield of detection of lymph node metastasis in thymic tumors, both allowing a better staging and a radical removal of the disease. LND, in fact, resulted in upstaging, respectively, 4.5 and 23% of patients with thymoma and thymic carcinoma, who had no preoperative evidence of LN metastasis. A similar finding had been previously described by Weksler et al. [6,7] who found that 84.6 and 80% of patients with thymic carcinoma and thymoma, respectively, were upstaged from lower stages because of node-positive status. A recent study by Song et al. [26] collected data from the SEER database on a total of 362 patients with TC and NETTs to analyze clinicopathologic factors that impact on patients’ prognosis, including intentional LND dissection. They found that the patients with positive regional lymph nodes had a shorter survival time than the negative ones (46 vs. 81 months; *p* = 0.004) and, interestingly, the patients who had undergone intentional LND had better OS than those who did not (90 vs. 50 months, *p* = 0.013). This is the first study assessing the role of surgical excision extended to lymph nodes in TC and NETTs to be of practical importance to predicting patients’ survival. Similarly, a recent analysis by Wang et al. [27] compared patients who had undergone LND with those who had not; results proved the N0 group to have a significant prognostic advantage over LND− (*p* = 0.018) and matched subgroup analyses confirmed N0 to be an independent positive prognostic factor compared with LND− (*p* = 0.006).

**Central message:** available data suggest that, along with its known capability of increasing the reliability of tumor staging by increasing the yield of detection of lymph node metastases in thymic tumors, LND can improve disease control, allowing a radical extirpation of the tumor itself.

### 4.3. Lymph Node Metastasis in Thymic Tumors: Who Is at Risk?

When analyzing the correlation of nodal involvement with other factors, the WHO histologic type, extent of primary tumor invasion (T) and surgical procedure with intentional N2 node dissection were found to be associated with increased positive lymph-nodes rates [2,22,23]. In their study, Hwang et al. [8] found that the rate of LN metastasis increased with T stage, starting from T2 (T1, 1%; T2, 50%; T3, 63.3%; *p* < 0.001). Since a significant correlation between the histologic subtype and the stage has been well documented [29], they analyzed the histologic subtypes and found that the rate of node metastasis in thymoma was significantly lower than in carcinoma (5.1% vs. 25%); it is notable that while type A, AB and B1 thymomas had no LN metastases, B2 and B3 thymomas showed no significant difference in the metastasis rate with carcinoma (*p* = 0.2). Moreover, tumor size and stage had proven to be closely related to each other, representing the biologic aggressiveness of the malignancy itself [29]; for this reason, when analyzing data about tumor size, they found that it was significantly larger in the node-metastasis group than in the node-negative one and that the optimal cutoff value for the prediction of node metastasis was 6 cm (metastasis rate 5% vs. 17.6%, *p* = 0.018). Furthermore, a study by Fang et al. [22] confirmed tumor histology and T category to be independent predictors of nodal involvement. Similar findings were those described by a retrospective study by Gu et al. [2] analyzing data from the ChART database: type A, AB and B1 thymomas proved to have no lymphatic metastasis, while in 1.3% of type B2 and B3 thymomas, 7.9% of thymic carcinomas and 16.7% of NETTs, metastases were detected. According to the pT stage, LN metastases rates were 0.2% in T1, 6.9% in T2, 8.5% in T3 and 7.4% in T4 (*p* < 0.001).

**Central message:** the available evidence indicates that patients with thymic malignancies should be preoperatively divided into high and low risk groups depending on tumor histology, tumor size and T category; therefore, systematic LND should be recommended for stage II or higher, WHO histology type B2/3, C, NETTs and tumor size > 6 cm.

### 4.4. Lymph Node Dissection in Thymic Tumors: N1, N2 or Both?

In the new proposed TNM classification, patients with N1 disease and without other metastases are classified as stage IVA, and those with N2 disease are classified as stage IVB [7]. Since no evidence has proven the necessity of N2 node harvesting, this is rarely included in the surgical resection of thymic tumors, unless performed intentionally. However, as previously said, N2 dissection turned out to be an important independent predictive factor for nodal disease [22,23]. Table 1 shows that in most tumors, LN metastases were located to N1 stations, while a lower rate of metastasis on N2 stations were found [3,4,22,23,25], proving the anterior mediastinal lymph nodes to be the primary site of lymphatic spread in thymic neoplasms. In a study by Kondo et al. [3], LN metastasis to N1 were seen in 90% of thymomas, 69% of carcinomas and 91% or NETTs; of these, 18% of thymomas, 44% of carcinomas and 60% of NETTs also metastasized to N2. Moreover, while thymoma (11%) and thymic carcinoid (9%) infrequently had skip metastases to N2, they were found in one third of thymic carcinomas. It goes without saying that while N1 could be considered the primary lymph node station in thymoma and NETTs, this could not be said for thymic carcinoma, which easily undergoes skip metastasis.

Murakami et al. [30] performed an anatomical study in 1990, proving that the right paratracheal node chain constituted the primary collecting vessel of the mediastinum and this element was later confirmed by different studies. Park et al. [4], in fact, proved that 30% of patients had metastasis to paratracheal lymph nodes exclusively to the right side; thus, they suggest a routine dissection of the anterior mediastinal lymph nodes and right paratracheal lymph nodes at a minimum. Similarly, Hwang et al. [8] detected 86% of the N2 node metastases in the right paratracheal lymph node group.

Fang et al. [22] found similar rates of N1 (4.7%) and N2 (2.9%) metastasis and 40% of the patients with nodal involvement had simultaneous N1/N2 disease. They also found that if N2 dissection must be performed, at least stations two and four on the right side and station seven on the left side should be harvested; moreover, according to the nomogram they built to predict nodal involvement, N2 nodal metastasis retrieval was higher for type B3-NETT and T3–4 tumors, and N2 involvement was usually on the ipsilateral side of tumor extension, except for NETTs.

**Central message:** in light of this evidence, the current recommendations by ITMIG and the European Society for Medical Oncology (ESMO) guidelines include a routine resection of the anterior mediastinal lymph nodes in all patients undergoing a thymectomy and removal of all suspected metastatic LN during the thymectomy; systematic sampling of deep lymph nodes (station two and four on the right side and station five on the left side) in locally advanced thymoma (stage III/IV) and a systematic resection of both N1 and N2 nodes in patients with thymic carcinoma and NETTs [31,32]. Moreover, recent studies suggest an LN dissection of the unique ipsilateral N2 nodes for thymomas and carcinomas; bilateral dissection is usually unnecessary, except for the rare cases of neuroendocrine tumors with a high prevalence of extensive nodal involvement [22].

### 4.5. Lymph Node Dissection in Thymic Tumors: How Many Nodes?

To date, no consensus has been reached regarding the extent of LND required to achieve optimal lymph node staging and the anatomic knowledge about lymph node metastasis in thymic neoplasms is still sparse. Most of the retrospective studies addressing this issue included cases in which at least one lymph node had been harvested [6,7]. In their study, Park et al. [4] dissected a mean number of 9.4 lymph nodes, thus defining 10 nodes as the division point between extensive or limited dissection. Results proved a better prognosis progressively for the N0b group (node negative by dissection of >10 lymph nodes), N0a group (node-negative by dissection of <10 lymph nodes) and N1 group (node-positive). The prognosis of the Nx group (no lymph node dissection) was similar to that of the N0a group, thus suggesting that the predictability of extensive lymph node dissection is more accurate, and that a limited lymph node dissection is insufficient.

**Central message:** available data are insufficient to suggest a recommendation on the number of nodes to be removed.

### 4.6. Lymph Node Dissection in Thymic Tumors: Does Surgical Approach Matter?

Current ITMIG guidelines suggest two surgical procedures for patients with thymic tumors with or without MG, respectively: *extended thymectomy* including the *en bloc* removal of the contiguous right and left mediastinal pleura, mediastinal and pericardiophrenic fatty tissues and dissection of aorta-pulmonary window in addition to complete thymectomy or *complete thymectomy* including the *en bloc* removal of the upper cervical poles and the surrounding mediastinal fat [33,34,35]. In all cases, complete resection (R0) of thymoma, thymic carcinoma and thymic neuroendocrine tumors is the most important aspect of treatment and the best independent prognostic factor. The advent of several mini-invasive techniques to accomplish thymoma resection, apart from the advantages of lower morbidity and pain, shorter hospitalization, faster patient recovery and reduced cost [35], raises the issue of being unilateral, not allowing for sampling of both sides of the chest. Few studies address the problem of the surgical approach and the few available pieces of evidence show no significant association between surgical approach and lymph node yield [22]. Moreover, it should be considered that most of the tumors selected for a mini-invasive approach are early stage lesions with tumor size < 5 cm [36] and theoretically, in cases of total thymectomy, N1 nodes would have already been included in the resection area. However, the mini-invasive approaches still guarantee the possibility of sampling N1 and N2 nodes, at least unilaterally, when needed for accurate lymph node staging.

**Central message:** the available data are insufficient to suggest a recommendation on the best surgical approach for thymic tumors; mini-invasive techniques, when appropriate, still guarantee LND of perithymic LN and sampling of N1 and N2 nodes, at least unilaterally, when needed for accurate lymph node staging.

### 4.7. Lymph Node Dissection in Thymic Tumors: Is There a Role for Adjuvant Treatment?

To date, lymph node dissection is of paramount importance in oncological surgery, since it increases the accuracy of tumor staging and the stratification of patients to plan the best tailored postoperative therapy and to provide better prediction of prognosis. Here comes the question whether the identification of nodal metastases could change the postoperative care of patients with thymoma. To date, only a few studies with few data address this issue and no evidence proves the efficacy of adjuvant treatments yet [4]. In a study by Park et al. [4], adjuvant therapy involving chemotherapy, radiotherapy or both was delivered to 73% of the population, in patients who had either undergone LND or not because adjuvant treatment was decided based on the extent of the tumor rather than by the lymph node metastasis. They found no local recurrences in all the cases. A similar experience was that by Weksler et al. [6,7], in which adjuvant radiotherapy was administered in 69.6% of node-positive patients with thymoma, registering an improvement in survival (145 vs. 62 months, *p* = 0.317), although it was not significant; adjuvant radiotherapy was also delivered to 62% of node-positive patients with thymic carcinoma and NETTs; also, in this case, survival was improved but the difference was not significant (60 vs. 42 months, *p* = 0.618). A recent study by Hwang et al. [23] described good outcomes (5-year overall survival: 82.4 and 54.9% for thymoma and thymic carcinoma, respectively) for node-positive patients treated with adjuvant chemo or radiotherapy. In thymic carcinoma lymph node-positive patients, the 5-year FFR rate was different, but not significant comparing the adjuvant treatment group with the non–adjuvant treatment group (23.5% vs. 32.8%, respectively; *p* = 0.64).

**Central message:** currently, no studies have specifically addressed postoperative adjuvant therapy in patients with stage IVB thymic malignancies with node metastases; consequently, no guidelines are available on the postoperative treatment of node-positive patients after surgical resection. Until further data are available, the role of adjuvant chemotherapy in completely resected thymic carcinoma is still vague, but radiation therapy seems to offer better survival rates.

There are some limitations of the present study. Firstly, as previously mentioned, the studies were very heterogeneous in describing lymph node dissection, in considering the different histological types and in the reporting of anatomical sites of nodal metastases. Moreover, few studies classified patients by T-stage and many of them used different classifications (Masaoka vs. TNMs) so that not all the data were easily comparable. Other limitations are the nature of the included studies and their geographical distribution; in fact, all the studies, but one, are retrospective, which leads to bias in data collection and patient selection and their geographical distribution is not fully representative, since most of the included patients are from China and Japan. To obtain reliable results, large prospective studies are needed.

## 5. Conclusions

Thymic tumors are the most common primary neoplasms of the anterior mediastinum, and their standard treatment is represented by radical thymectomy with *en bloc* resection of the surrounding thymic tissue. Due to the rarity of the LN involvement, lymph node dissection is not considered as a standard procedure in thymic cancer surgery and the role of LND remains controversial. Even if they are still few, the current pieces of evidence suggest that lymph node metastases have higher incidence rates in TC and NETTs rather than in thymoma. A systematic LND should be recommended for stage II or higher, WHO histology type B2/3, C, NETTs and tumor size > 6 cm since in these cases it can improve disease control, allowing a radical extirpation of the tumor itself.

## Figures and Tables

**Table 1:** Studies comparing data about lymph node metastasis in thymic tumors over the last 20 years.

Author	Year	Country	P/R	N pts	Intentional Lymphadenectomy	n LN Dissected (Mean)	Type of Tumor (%)			Lymph Node Metastasis (%)				Metastatic Stations (%)	
							T	TC	NETT	Overall	T	TC	NETT	N1	N2
Kondo [3]	2003	Japan	R	1320	No	-	82.8	14.1	3.1	6.7	1.8	26.8	27.5	-	-
Park [4]	2013	Korea	R	29	Yes	9.4	-	100	-	20.8	-	20.8	-	-	-
Weissferdt [20]	2012	USA	R	33	Yes	-	-	100	-	37	-	37	-	16.7	20
Weksler [6]	2015	USA	R	229	No	3	-	76.9	23.1	40.2	-	33.5	62.3	-	-
Weksler [7]	2015	USA	R	442	No	2	100	-	-	13.3	13.3	-	-	-	-
Hwang [8]	2016	Korea	R	131	Yes	10.3	75.6	24.4	-	9.9	5.1	25	-	4.6	5.4
Gu [2]	2017	China	R	1617	No	-	81	16.4	2.6	2.2	0.5	7.9	16.7	-	-
Zhao [21]	2017	China	R	116	Yes	2.3 (TC) 1.8 (NETT)	-	81.8	18.1	53.4	-	48.4	76.2	50	35.5
Fang [22]	2018	China	P	275	Yes	4.9	88.4	8.7	2.9	5.5	2.1	25	50	4.7	2.9
Hwang [23]	2018	Korea	R	446	Yes	8.9	66.8	33.2	-	15.0	6.7	31.8	-	9.6	5.4
Wen [24]	2018	China	R	3947	No	-	70.6	21.9	7.4	6.1	11.1	33.0	57.0	-	-
Cheufou [25]	2019	Germany	R	53	No	3.5	-	81.1	18.9	30.2	-	24.5	5.6	20.8	9.4
Song [26]	2019	China	R	363	No	-	-	66.3	33.7	34.3	-	-	-	-	-
Wang [27]	2020	USA	R	398	Yes	1–3 (46%) >4 (47%)	-	78.6	21.4	36.2	-	30	58.8	-	-
Clermidy [28]	2021	France	R	54	Yes	3.9	100	-	-	5.6	5.6	-	-	0	6.1

P/R: prospective/retrospective; T: thymoma; TC: thymic carcinoma; NETT: thymic neuroendocrine tumor; LN: lymph node.

**Table 2:** Studies comparing survival rates in N0 vs. N+ patients over the last 20 years. Difference of values in **bold** is statistically significant.

Author	N pts	Type of Tumor (%)			OS			
		T	TC	NETT		Overall (mo)	5Y (%)	10Y (%)
Weksler [6]	229	-	76.9	23.1	N0	**124**		
					N+	**47**		
Weksler [7]	442	100	-	-	N0	**144**	**79**	**32**
					N+	**98**	**66**	**16**
Gu [2]	1617	81	16.4	2.6	N0			**92.5**
					N+			**51.9**
Song [26]	362	-	66.3	33.7	N0	**81**		
					N+	**46**		
Wang [27] *	398	-	78.6	21.4	N0	**86**		**37**
					N+	**41**		**6**

* Intentional lymphadenectomy; T: thymoma; TC: thymic carcinoma; NETT: thymic neuroendocrine tumor; OS: overall survival; 5Y: five years survival rate; 10Y: ten years survival rate.

**Table 3:** Studies comparing the incidence of lymph node metastasis in thymic tumors according to the T-stage over the last 20 years. Difference of values in **bold** is statistically significant.

Author	N pts	Type of Tumor (%)		Incidence of Nodal Metastasis According to T Stage (%)			
				T1	T2	T3	T4
Park [4] *	37	TC	100	**0**	**0**	**35.3**	
Gu [2]	1617	T	81	**0.2**	**6.9**	**8.5**	**7.4**
		TC	16.4				
		NETT	2.6				
Zhao [21]	343	TC	81.9	33.3	28.6	53.4	
		NETT	18.1	50	66.7	90.9	
Fang [22] *	275	T	88.4	**2.7**	**7.7**	**18.4**	**50**
		TC	8.7				
		NETT	2.9				
Cheufou [25]	53	TC	81.1	6.25	6.25	37.5	50
		NETT	18.9				

* Intentional lymphadenectomy; T: thymoma; TC: thymic carcinoma; NETT: thymic neuroendocrine tumor.

## Supplementary Materials

The following are available online at https://www.mdpi.com/article/10.3390/cancers13205085/s1, **Table S1**: Quality assessment of studies reporting on the role of LND in patients with thymic cancers according to the National Heart, Lung and Blood Institute criteria. PRISMA 2020 Main Checklist.
