# Supplementary material for: Lymph Nodes Involvement and Lymphadenectomy in Thymic Tumors: Tentative Answers for Unsolved Questions
Source: Cancers (Basel). 2021 Oct 11;13(20):5085. doi: 10.3390/cancers13205085 (PMC8534239; doi:10.3390/cancers13205085)
Supplement: Supplementary file 1 [file cancers-13-05085-s001.zip › Table S1.pdf]

Table S1. Quality assessment of studies reporting on the role of LND in patients with thymic cancers according to the National Heart, Lung, and Blood Institute criteria.

	**Kondo [3]**			**Park [4]**			**Weissferdt [19]**			**Weksler [6]**			**Weksler [7]**			**Hwang [8]**		
**Criteria**	Yes	No	Other	Yes	No	Other	Yes	No	Other	Yes	No	Other	Yes	No	Other	Yes	No	Other
1. Was the research question or objective in this paper clearly stated and appropriate?	X			X				X		X			X			X		
2. Was the study population clearly specified and defined?	X			X			X			X			X			X		
3. Did the authors include a sample size justification?		X			X			X			X			X			X	
4. Were controls selected or recruited from the same or similar population that gave rise to the cases (including the same timeframe)?	X					X		X		X			X			X		
5. Were the definitions, inclusion and exclusion criteria, algorithms or processes used to identify or select cases and controls valid, reliable, and implemented consistently across all study participants?		X		X					X		X			X		X		
6. Were the cases clearly defined and differentiated from controls?		X				X			X			X			X	X		
7. If less than 100 percent of eligible cases and/or controls were selected for the study, were the cases and/or controls randomly selected from those eligible?		X			X				X			X			X			X
8. Was there use of concurrent controls?		X			X			X		X			X			X		
9. Were the investigators able to confirm that the exposure/risk occurred prior to the development of the condition or event that defined a participant as a case?	X					X		X				X			X			X
10. Were the measures of exposure/risk clearly defined, valid, reliable, and implemented consistently (including the same time period) across all study participants?			X	X				X	X			X			X	X		
11. Were the assessors of exposure/risk blinded to the case or control status of participants?		X			X			X			X			X			X	
12. Were key potential confounding variables measured and adjusted statistically in the analyses? If matching was used, did the investigators account for matching during study analysis?		X		X				X		X			X			X		
Quality rating	POOR			FAIR			POOR			FAIR			FAIR			GOOD		

	**Gu [2]**			**Zhao [20]**			**Fang [21]**			**Hwang [22]**			**Wen [23]**			**Cheufou [24]**		
**Criteria**	Yes	No	Other	Yes	No	Other	Yes	No	Other	Yes	No	Other	Yes	No	Other	Yes	No	Other
1. Was the research question or objective in this paper clearly stated and appropriate?	X			X			X			X			X			X		
2. Was the study population clearly specified and defined?	X			X			X			X			X			X		
3. Did the authors include a sample size justification?		X			X			X			X			X			X	
4. Were controls selected or recruited from the same or similar population that gave rise to the cases (including the same timeframe)?	X					X			X	X			X				X	
5. Were the definitions, inclusion and exclusion criteria, algorithms or processes used to identify or select cases and controls valid, reliable, and implemented consistently across all study participants?	X			X			X			X			X			X		
6. Were the cases clearly defined and differentiated from controls?			X			X			X	X			X				X	
7. If less than 100 percent of eligible cases and/or controls were selected for the study, were the cases and/or controls randomly selected from those eligible?			X			X			X			X			X		X	
8. Was there use of concurrent controls?		X			X			X		X			X				X	
9. Were the investigators able to confirm that the exposure/risk occurred prior to the development of the condition or event that defined a participant as a case?			X		X			X				X			X		X	
10. Were the measures of exposure/risk clearly defined, valid, reliable, and implemented consistently (including the same time period) across all study participants?	X			X			X			X			X				X	
11. Were the assessors of exposure/risk blinded to the case or control status of participants?		X			X			X			X			X			X	
12. Were key potential confounding variables measured and adjusted statistically in the analyses? If matching was used, did the investigators account for matching during study analysis?	X					X			X	X			X					X
Quality rating	FAIR			POOR			POOR			GOOD			GOOD			POOR		

	Song [25]			Wang [26]			Clermidy [27]		
**Criteria**	Yes	No	Other	Yes	No	Other	Yes	No	Other
1. Was the research question or objective in this paper clearly stated and appropriate?	X			X			X		
2. Was the study population clearly specified and defined?	X			X			X		
3. Did the authors include a sample size justification?		X			X			X	
4. Were controls selected or recruited from the same or similar population that gave rise to the cases (including the same timeframe)?		X		X			X		
5. Were the definitions, inclusion and exclusion criteria, algorithms or processes used to identify or select cases and controls valid, reliable, and implemented consistently across all study participants?			X			X	X		
6. Were the cases clearly defined and differentiated from controls?			X			X	X		
7. If less than 100 percent of eligible cases and/or controls were selected for the study, were the cases and/or controls randomly selected from those eligible?			X			X	X		
8. Was there use of concurrent controls?		X			X		X		
9. Were the investigators able to confirm that the exposure/risk occurred prior to the development of the condition or event that defined a participant as a case?			X			X			X
10. Were the measures of exposure/risk clearly defined, valid, reliable, and implemented consistently (including the same time period) across all study participants?			X			X	X		
11. Were the assessors of exposure/risk blinded to the case or control status of participants?		X			X			X	
12. Were key potential confounding variables measured and adjusted statistically in the analyses? If matching was used, did the investigators account for matching during study analysis?	X			X			X		
Quality rating	POOR			FAIR			GOOD		
